# Immuno-PET Imaging of Tumour PD-L1 Expression in Glioblastoma

**DOI:** 10.3390/cancers15123131

**Published:** 2023-06-09

**Authors:** Gitanjali Sharma, Marta C. Braga, Chiara Da Pieve, Wojciech Szopa, Tatjana Starzetz, Karl H. Plate, Wojciech Kaspera, Gabriela Kramer-Marek

**Affiliations:** 1 Division of Radiotherapy and Imaging, Institute of Cancer Research, London SW7 3RP, UK; 2 Department of Neurosurgery, Medical University of Silesia, 41-200 Sosnowiec, Poland; 3 Edinger Institute, Institute of Neurology, 60528 Frankfurt, Germany; 4 German Consortium for Translational Cancer Research, DKTK, 69120 Heidelberg, Germany

**Keywords:** immuno-PET, PD-L1, glioblastoma, affibody molecule

## Abstract

**Simple Summary:**

Almost all patients with glioblastoma (GBM) eventually relapse, mainly due to adaptive and acquired resistance that results from tumour heterogeneity and its relatively immune-depleted (“cold”) microenvironment. High levels of programmed death ligand-1 (PD-L1) have been associated with GBM invasiveness and immuno-resistance. Presently, there is no standardised approach for the assessment of PD-L1 expression level that would help in predicting the response to immune checkpoint inhibitors. Therefore, we investigated the ability of a radiolabelled Z_PD-L1_ affibody molecule to measure the expression level of PD-L1 in GBM xenograft models.

**Abstract:**

There is no established method to assess the PD-L1 expression in brain tumours. Therefore, we investigated the suitability of affibody molecule (Z_PD-L1_) radiolabelled with F-18 (Al^18^F) and Ga-68 to measure the expression of PD-L1 in xenograft mouse models of GBM. Mice bearing subcutaneous and orthotopic tumours were imaged 1 h post-radioconjugate administration. Ex vivo biodistribution studies and immunohistochemistry (IHC) staining were performed. Tumoural PD-L1 expression and CD4+/CD8+ tumour-infiltrating lymphocytes were evaluated in human GBM specimens. Z_PD-L1_ was radiolabelled with radiochemical yields of 32.2 ± 4.4% (F-18) and 73.3 ± 1.8% (Ga-68). The cell-associated radioactivity in vitro was consistent with PD-L1 expression levels assessed with flow cytometry. In vivo imaging demonstrated that ^18^F-AlF-NOTA-Z_PD-L1_ can distinguish between PD-L1 high-expressing tumours (U87-MGvIII) and PD-L1-negative ones (H292_PD-L1Ko_). The radioconjugate was quickly cleared from the blood and normal tissues, allowing for high-contrast images of brain tumours as early as 1 h post-injection. ^68^Ga-NOTA-Z_PD-L1_ showed heterogeneous and diffuse accumulation that corresponded to the extensively infiltrating GCGR-E55 tumours involving contiguous lobes of the brain. Lastly, 39% of analysed GBM patient samples showed PD-L1+ staining of tumour cells that was associated with elevated levels of CD4+ and CD8+ lymphocytes. Our results suggest that the investigated radioconjugates are very promising agents with the potential to facilitate the future design of treatment regimens for GBM patients.

## 1. Introduction

Glioblastoma (GBM) is a high-grade malignant brain tumour that accounts for about 15% of all primary brain tumours [1]. Despite some advances in treatment regimens, the prognosis of GBM patients remains extremely poor. The median overall survival (OS) time is between 12–15 months, and the 5-year survival rate is <10% [2]. Compared to other tumours, GBMs are especially hard to treat. The infiltrative nature of these tumours prevents complete surgical resection; the use of external beam radiotherapy is limited by radiation-induced neurotoxicity, and the systemic delivery of therapeutic agents is inhibited by the restrictive nature of the blood-brain barrier (BBB). Unsurprisingly, most patients become refractory to treatment and succumb to disease, presenting an urgent unmet clinical need for novel treatment paradigms that will provide more durable remissions [3,4]. 

Immune checkpoint inhibitors (ICPIs) targeting either the programmed cell death protein 1 (PD-1, e.g., nivolumab, pembrolizumab) or its ligand (PD-L1; e.g., durvalumab, atezolizumab) have recently shown promising efficacy across several tumour types [5]. Data from clinical trials revealed that PD-L1 expression measured with tumour proportion score (TPS) or combined pathological score (CPS) is a potential biomarker to guide the selection of patients who could benefit from ICPIs [6]. Regrettably, for GBM patients, only modest and unpredictable responses to ICPIs have been reported so far. This is most likely due to an immune-suppressive GBM microenvironment characterised by: (i) absence of tumour-infiltrating lymphocytes (TILs); (ii) exhaustion of cytotoxic T-lymphocytes (CTLs); and (iii) high levels of immunosuppressive cytokines [7]. Encouragingly, recent findings have demonstrated that pronounced infiltration of pre-existing CD8+ CTLs into the tumour microenvironment can render GBM more responsive to ICPIs. Cloughesy et al. have shown that neoadjuvant administration of PD-1 blockade combined with surgical treatment activated local and systemic immune responses in patients with recurrent GBM [8]. Furthermore, high PD-L1 expression has been associated with greater invasiveness and aggressiveness of GBM cells. Interestingly, tumours with mesenchymal features seem to have elevated levels of PD-L1 and the highest presence of tumour-associated macrophages (TAMs), CD8+, CD3+ and FOXP3+T cells, which suggests they may be more immunoreactive by nature and therefore more sensitive to treatment with checkpoint inhibitors [9].

Thus, an accurate measurement of the PD-L1 expression level in tumours is of paramount importance for more accurate patient stratification and treatment selection. Currently, PD-L1 testing is performed mainly using immunohistochemistry (IHC), using samples dissected postoperatively or via biopsy. Providing just a limited snapshot of the whole tissue, IHC might not fully reflect the protein status in the entire tumour mass, leading to an increased number of false-positive/-negative results [10,11]. There is growing evidence demonstrating that immuno-positron emission tomography (immuno-PET) can provide a real-time quantitative readout of PD-L1 expression level with the potential for sequential measurements that facilitate monitoring of dynamic changes in PD-L1 expression without the need for repeated biopsies [12]. Importantly, Bensch et al. have recently reported an increased uptake of zirconium-89-labelled atezolizumab (^89^Zr-DFO-Atezolizumab) in lymphoid tissue and sites of inflammation in 22 patients with a variety of tumour types. Furthermore, the heterogeneity of uptake in tumour masses and the baseline (pretreatment) PET signals correlated with the response to atezolizumab treatment [13]. Along the same line, Nienhuis et al. have shown the accumulation of an adnectin-based small molecule radiotracer (^18^F-BMS986192) targeting PD-L1 in intra- and extracerebral metastatic lesions [14]. 

Long-lived PET isotopes, such as zirconium-89 (t_1/2_ = 78.4 h) or iodine-124 (t_1/2_ = 108 h), are used to radiolabel full-size mAbs (t_1/2_ = 10–21 days in vivo) due to the compatibility between the physical and biological half-lives of the radionuclides and the biomolecules, respectively [15]. Unfortunately, the slow body clearance and prolonged serum half-life of the radiolabelled mAbs may provide relatively high radiation doses for the patients [16]. Conversely, due to their small size (ca. 58 amino acid residues, ca.7 kDa), affibody binders have fast blood clearance and therefore can be labelled with widely available short-lived radioisotopes, such as fluorine-18 (t_1/2_ = 108 min) or gallium-68 (t_1/2_ = 68 min). Several studies, including ours, have previously described that the robust structure of affibody molecules against different targets (e.g., EGFR, HER2, HER3) can tolerate harsh radiolabelling conditions (e.g., temperatures up to 95 °C, high/low pH and vigorous shaking) without reducing their target-binding capacities [17,18,19,20]. Furthermore, we demonstrated that the EGFR-specific affibody radioconjugate ^18^F-AlF-NOTA-Z_EGFR:03115_ could target brain tumours, allowing for a clear GBM tumour delineation as early as 1 h postinjection [21]. Herein, we describe the use of Z_PD-L1_, a PD-L1-targeting affibody molecule, to noninvasively measure PD-L1 expression level in orthotopic xenograft GBM models with immuno-PET.

## 2. Materials and Methods

### 2.1. General

All the reagents and solvents were purchased from commercial sources. Detailed information about the reagents and isotopes is given in the Supplemental Data. NOTA-Z_PD-L1_, prepared by attaching NOTA-maleimide to the terminal cysteine on the affibody molecule against human PD-L1 Z_PD-L1_, was kindly provided by GE Healthcare Limited (London, UK).

### 2.2. Preparation of ^18^F-AlF-NOTA-Z_PD-L1_ and ^68^Ga-NOTA-Z_PD-L1_

The conjugation of NOTA-Z_PD-L1_ and the consequent ^18^F and ^68^Ga radiolabelling procedures are described in the Supplemental Data.

### 2.3. Cell Lines and Tumours

Human non-small cell lung cancer cell lines (H292 and H292_PD-L1KO_) were provided by the Medical Oncology Laboratory, University Medical Centre, Groningen (UMCG). The human glioblastoma (U87-MGvIII) cell line was provided by Dr Frank Furnari (Ludwig Cancer Research, San Diego, CA, USA). The glioma stem cell line (GCGR-E55) was provided by Prof. Steven Pollard (University of Edinburgh, UK). The cells were grown as described in the Supplemental Data. TCGA RNA Seq data were obtained from the TCGA Data Portal (http://cancergenome.nih.gov). Tissue specimens from the patients with newly diagnosed GBM (n = 36) were collected for IHC in accordance with the protocol approved by the Institutional Bioethical Committee of Medical University of Silesia (Katowice, Poland). 

### 2.4. Flow Analysis of PD-L1 Expression

To assess the PD-L1 expression in H292, U87-MGvIII, GCGR-E55 and H292_PD-L1KO_ cell lines, the cells were washed once with cold PBS and incubated for 40 min at 4 °C with either a specific anti-CD274 PD-L1 antihuman antibody (MIH1, PE Cyanine-7, 25-5983-42, Invitrogen, Waltham, MA, USA) or PBS (for the unstained controls). Flow cytometry was performed using a BD LSRII flow cytometer (BD, Biosciences, Swindon, UK) and analysed using FlowJo v10 (BD, Biosciences). More details about the assay are described in the Supplemental Data.

### 2.5. ^18^F-AlF-NOTA-Z_PD-L1_ and ^68^Ga-NOTA-Z_PD-L1_ In Vitro Studies 

Specificity of binding: For this study, H292, U87-MGvIII, GCGR-E55 and H292_PD-L1KO_ cells were seeded 48 h prior to the experiment. On the following day, one group of cells was stimulated with human interferon gamma (IFNγ, 20 ng/mL, PHC4031, Gibco, Thermo Fisher Scientific, Waltham, MA, USA) overnight whilst the rest (control cells) were incubated with their respective media only. Afterwards, cells were incubated with a 5 nM solution of either ^18^F-AlF-NOTA-Z_PD-L1_ or ^68^Ga-NOTA-Z_PD-L1_. For blocking experiments, cells were pre-incubated with a 100-fold molar excess of nonlabelled affibody conjugate (NOTA-Z_PD-L1_. After 1 h, cell-associated radioactivity was determined with γ-counter (Wizard^2^ 2480, PerkinElmer, Beaconsfield, UK). The data were expressed as a percentage of the incubated dose (%ID) per mg of the protein lysate. A detailed description of the procedures is given in the Supplemental Data.

Saturation radioligand binding assay: To determine the dissociation constant (K_d_) of ^18^F-AlF-NOTA-Z_PD-L1_, H292 cells were seeded 24 h prior to this study. On the following day, cells were incubated with increasing concentrations of ^18^F-AlF-NOTA-Z_PD-L1_ (0.01–15 nM) for 1 h at 4 °C. Nonspecific binding was determined through co-incubation of 100-fold molar excess of non-radiolabelled affibody conjugate. The cells were then rinsed and collected for radioactivity measurement with γ-counter. The K_d_ was estimated by plotting the amount of bound (nM) vs. free radioconjugate ligand (nM). The assay is described in detail in the Supplemental Data.

### 2.6. Mouse Models

The detailed methods are described in the Supplemental Data. All experiments were performed in compliance with the licence issued under the UK Animals (Scientific Procedures) Act 1986, the UK National Cancer Research Institute Guidelines for Animal Welfare in Cancer Research [22] and the ARRIVE guidelines for reporting animal research. All experiments were conducted under the Project Licence PPL PCC916B22 and approved by the UK Home Office and by the local ethical review committee. Female nude mice (crl:NU(NCR)-Fox1n1nu) and female NSG (NOD.Cg-Prkdc^scid^ II2rgtm1Wjl/SzJ) were obtained from Charles River Laboratories (Harlow, UK). For subcutaneous models, animals were injected with U87-MGvIII cells or H292_PD-L1KO_ cells. For orthotopic implantation, animals were stereotactically injected with U87-MGvIII or GCGR-E55 cells Orthotopic tumour growth was monitored with the 1 T M3™ MRI system (Aspect Imaging, Shoham, Israel). The growth curve of the GCGR-E55 model is shown in Supplementary Figure S1. Subcutaneous tumour growth was monitored through calliper measurements. More experimental details are given in the Supplemental Data.

### 2.7. ^18^F-AlF-NOTA-Z_PD-L1_ and ^68^Ga-NOTA-Z_PD-L1_ In Vivo Imaging and Ex Vivo Studies 

For PET studies, animals were anaesthetised and injected with ^18^F-AlF-NOTA-Z_PD-L1_ (0.5 µg, 0.1 ± 0.05 MBq/mouse; or 1 µg, 0.4 ± 0.3 MBq/mouse) intravenously via the tail vein. For the brain PET imaging studies, mice were either injected with ^18^F-AlF-NOTA-Z_PD-L1_ (1 µg, 0.2–0.76 MBq/mouse) or ^68^Ga-NOTA-Z_PD-L1_ (1 µg, 0.73–1.55 MBq/mouse) intravenously via the tail vein. For blocking studies, animals were co-injected with 400 µg of the nonlabelled affibody molecule. PET/CT scans were acquired 1 h postinjection using a small-animal PET scanner (Albira PET/SPECT/CT, Bruker, MA, USA). Images were acquired as stated in the Supplemental Data. Image analysis was performed using the PMOD software package (PMOD Technologies Ltd., Zurich, Switzerland). Radioactivity uptake in the tumour was quantified using volume-of-interest (VOI) analysis and expressed as the mean (Mean) and the mean of the 50 hottest voxels (Mean_50_) within the VOI. Data were expressed as the percentage of the injected dose per gram of tissue (%ID/g) normalised to a calibration factor (MBq/g/counts) calculated by scanning a source (^18^F or ^68^Ga) of known activity and volume.

For biodistribution studies, blood and major organs, as well as the tumours, were collected and weighed, and the radioactive content was measured with γ-counting. The decay-corrected data were expressed as a percentage of injected dose per gram of tissue (%ID/g) (n = 4 ± SD).

### 2.8. Ex Vivo Immunohistochemistry 

Formalin-fixed brain and tumour tissues were embedded in paraffin, sectioned and mounted on microscope slides. The detailed staining procedures with the various antibodies are described in the Supplemental Data.

### 2.9. Statistical Analysis 

Statistical analysis for the in vivo data was performed using ordinary one-way ANOVA with Tukey’s multiple comparisons tests, and significance was considered for * *p <* 0.05, ** *p <* 0.01, *** *p <* 0.001 and **** *p <* 0.0001. Correlation between PET imaging and biodistribution data was performed with simple linear regression. The chi-square (χ^2^) test was used to determine the statistical relationship between CD4+ and CD8+ cells distribution (high/low) and PD-L1 protein status (positive/negative). All analyses were carried out with Prism Software (Graphpad Software v9.1.1, San Diego, CA, USA). 

## 3. Results

### 3.1. Radiolabelling 

**Figure 1:**
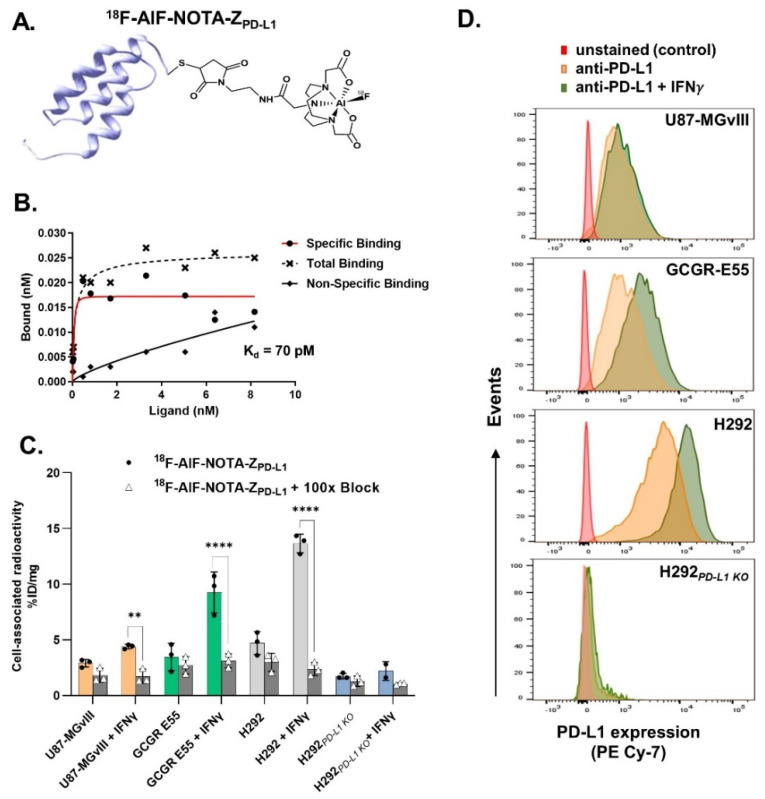
(**A**) Schematic illustrating the fluorine-18-radiolabelled affibody molecule structure. (**B**) Saturation binding of ^18^F-AlF-NOTA-Z_PD-L1_ to H292 highly PD-L1-positive cells. (**C**) In vitro binding specificity of ^18^F-AlF-NOTA-Z_PD-L1_ (5 nM), with and without IFN γ stimulation, as well as blocking (n = 3). **, *p* = 0.0029, ****, *p* ≤ 0.0001. (**D**) Histograms showing expression levels of PD-L1 in a panel of cell lines (as assessed using flow cytometry with a PE Cyanine-7-stained PD-L1 antibody).

The PD-L1-specific affibody conjugate NOTA-Z_PD-L1_ was successfully radiolabelled with fluorine-18 (t_1/2_ = 108 min) and gallium-68 (t_1/2_ = 68 min). Both radioconjugates were achieved with a >98% radiochemical purity (RCP) (Supplementary Figures S2–S4) and a decay-corrected radiochemical yield (RCY) of 32.2 ± 4.4% and 73.3 ± 1.8% for ^18^F-AlF-NOTA-Z_PD-L1_ and ^68^Ga-NOTA-Z_PD-L1_, respectively. The apparent molar activity at the end of synthesis was 4.8 ± 1.6 MBq/nmol (apparent specific activity = 0.67 ± 0.23 MBq/µg) and 13.5 ± 2.6 MBq/nmol (apparent specific activity = 1.84 ± 0.3 MBq/µg) for ^18^F-AlF-NOTA-Z_PD-L1_ and ^68^Ga-NOTA-Z_PD-L1_, respectively. The schematic structures of the radioconjugates are shown in Figure 1A and 5A. 

### 3.2. In Vitro Evaluation of the Radioconjugates

The dissociation constant (K_d_) of ^18^F-AlF-NOTA-Z_PD-L1_ was determined with a cell-based saturation assay using PD-L1 highly expressing H292 cells. The K_d_ was measured to be 0.070 ± 0.014 nM (Figure 1B). The calculated B_max_ was 276,000 sites/cell (46,000 fmol/mg).

The PD-L1-binding specificity of both ^18^F-AlF-NOTA-Z_PD-L1_ and ^68^Ga-NOTA-Z_PD-L1_ was assessed using cell lines with different PD-L1 statuses, showing that the cell-associated radioactivity was consistent with the PD-L1 protein expression levels in each cell line (Figure 1C).

Moreover, increased and PD-L1-dependent binding was observed when the cells were subjected to IFNγ stimulation compared to the nonstimulated controls (Figure 1C and 5B). The cell-associated radioactivity signals were also in line with the PD-L1 expression levels confirmed with flow cytometry analysis (Figure 1D). Finally, pre-incubating the cells with a 100-fold molar excess of nonlabelled affibody molecule resulted in significantly reduced radioactivity signals, further confirming the radioconjugate’s specificity (Figure 1C). 

### 3.3. PD-L1-Specific Radioconjugates Accumulation In Vivo

**Figure 2:**
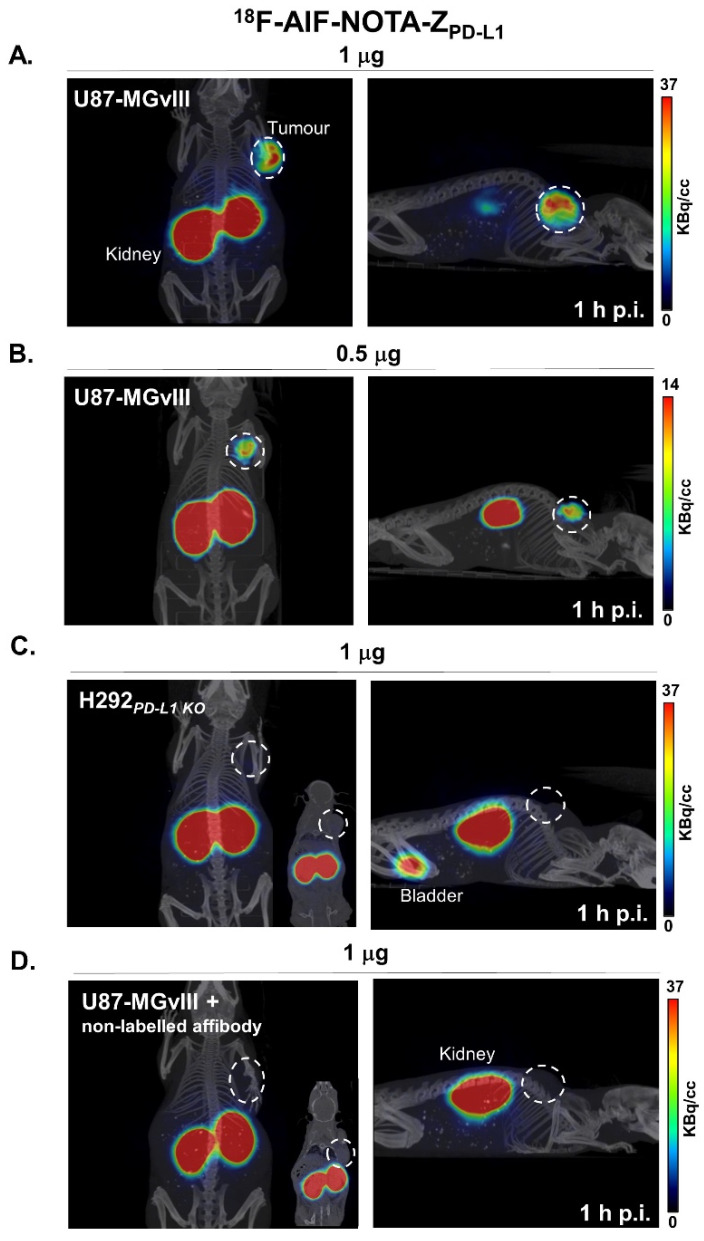
Representative whole-body PET/CT images acquired 1 h after ^18^F-AlF-NOTA-Z_PD-L1_ administration: (**A**) 1 µg dose of radiolabelled affibody in subcutaneous PD-L1+ U87-MGvIII tumour (0.4 ± 0.3 MBq/mouse, n = 4). (**B**) 0.5 µg dose in subcutaneous PD-L1+ U87-MGvIII tumour (0.1 ± 0.05 MBq/mouse, n = 4). (**C**) 1 µg dose in PD-L1− tumour model, H292_PD-L1KO_ (0.4 ± 0.3 MBq/mouse, n = 4). (**D**) 1 µg dose spiked with 400-fold excess of nonlabelled affibody molecule (0.4 ± 0.3 MBq/mouse, n = 4).

Although athymic nude mice lack a thymus and are unable to produce T cells, large quantities of IFNγ are still secreted by the present NK cells and other remaining immune cells, which might robustly stimulate PD-L1 expression in tumour cells. Therefore, the in vivo specificity of ^18^F-AlF-NOTA-Z_PD-L1_ was evaluated in PD-L1-expressing (U87-MGvIII) and PD-L1-negative (H292_PD-L1KO_) tumour-bearing mice. PET imaging studies showed that ^18^F-AlF-NOTA-Z_PD-L1_ accumulates in the subcutaneous PD-L1+ U87-MGvIII tumours from 1 h postinjection (Figure 2A,B). Two different quantities of the radioconjugate (0.5 and 1 µg) were administered to investigate the effect of the injected protein dose on biodistribution and tumour uptake. There was no significant difference in uptake in normal organs between these two injected doses, and although the average tumour uptake was 6.5 ± 0.9%ID/g and 3.9 ± 0.3%ID/g for 1 µg and 0.5 µg, respectively (*p* < 0.01, Figure 2A,B), the tumours were clearly visualised in both cases. Furthermore, radioconjugate accumulation was significantly higher in PD-L1-expressing U87-MGvIII tumours than in PD-L1-negative H292_PD-L1KO_ (6.5 ± 0.9%ID/g vs. 0.7 ± 0.2%ID/g, *p* < 0.0001, Figure 2C). Additionally, the co-injection of 400-fold excess of nonlabelled affibody molecule reduced the tumour uptake significantly (0.6 ± 0.2%ID/g, *p* < 0.0001, Figure 2D), further confirming the specificity of the radiotracer in vivo. 

**Figure 3:**
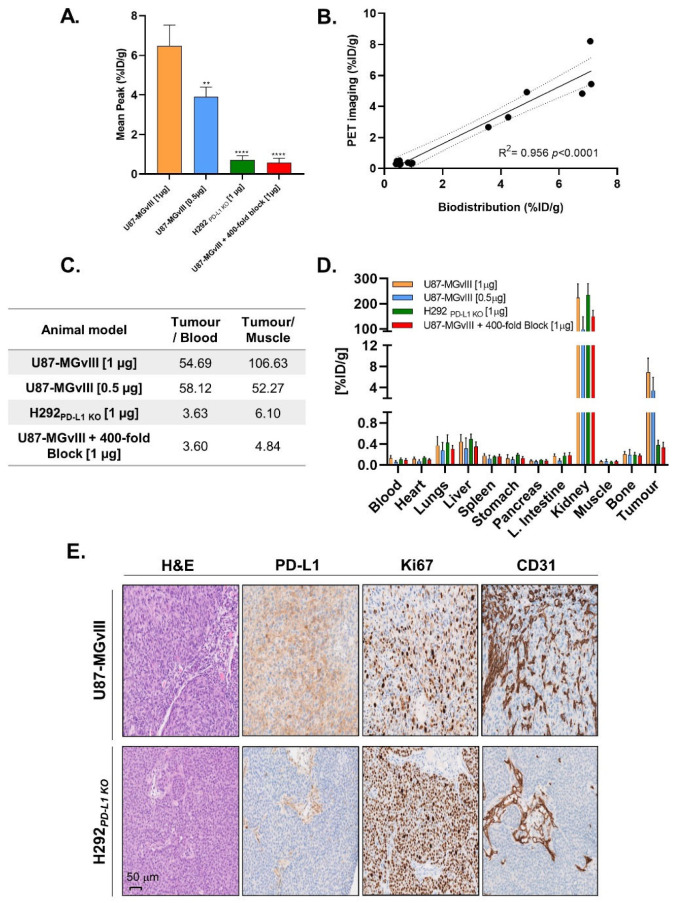
(**A**) PET imaging quantified as %ID/g_Mean_. **, *p* = 0.0012. ****, *p* ≤ 0.0001. (**B**) PET to biodistribution correlation. (**C**) Associated tumour/blood and tumour/muscle ratios. (**D**) Ex vivo biodistribution after injection of two doses of ^18^F-AlF-NOTA-Z_PD-L1_ (1 µg and 0.5 µg). (**E**) H&E staining and IHC analysis of PD-L1, Ki67 and CD31.

The tumour uptake quantification of ^18^F-AlF-NOTA-Z_PD-L1_ determined with PET (Figure 3A) was in line with ex vivo biodistribution studies performed right after the imaging (R^2^ = 0.956, *p* < 0.0001, Figure 3B). Since Z_PD-L1_ does not have cross-reactivity with the murine PD-L1 counterpart, the contrast between tumour and normal organs might be different in humans or humanised mice models. Nevertheless, the calculated tumour-to-blood and tumour-to-muscle ratios for this study were 55 and 107 for 1 µg of Z_PD-L1_ and 53 and 58 for 0.5 µg of Z_PD-L1_ (Figure 3C). The distribution of the radioconjugate in major organs is depicted in Figure 3D and Figure S5. As expected, among the nontargeted organs, the highest radioactivity signal was measured in the kidney (98–234%ID/g, Figure 3D), which was due to the renal excretion of the affibody molecule that filtered through the glomerular membrane and underwent reabsorption in proximal tubules in the kidney [23]. Importantly, tumour targeting with the ^18^F-AlF-NOTA-Z_PD-L1_ correlated with PD-L1 immunohistochemical staining (Figure 3E). Of note, both U87-MGvIII and H292_PD-L1KO_ tumours were enriched in proliferative cells, as displayed with enhanced staining of the Ki67 marker that was confined to the nucleus. Furthermore, CD31 staining showed no discernible difference in vessel density between these two models, indicating the preferential radioconjugate uptake in U87-MGvIII was unlikely to relate to different degrees of vascularisation (Figure 3E).

### 3.4. Targeting PD-L1 in the Brain

**Figure 4:**
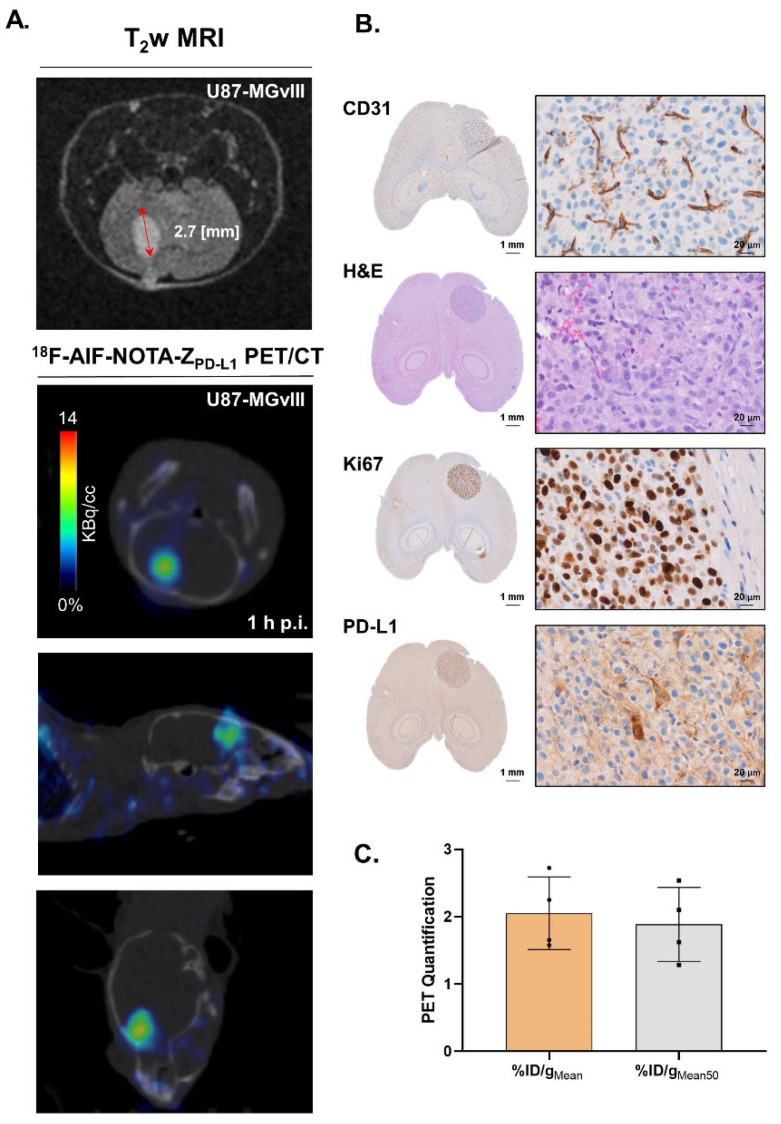
(**A**) PET/CT and T_2_w MRI scans of intravenous injections of ^18^F-AlF-NOTA-Z_PD-L1_ 1 h postinjection in U87-MGvIII orthotopic tumours. The mice received 0.2–0.76 MBq of radioconjugate (n = 4) and (**B**) associated IHC staining. (**C**) PET quantification of ^18^F-AlF-NOTA-Z_PD-L1_ tumour uptake expressed as %ID/g_Mean_ and %ID/g_Mean50_.

**Figure 5:**
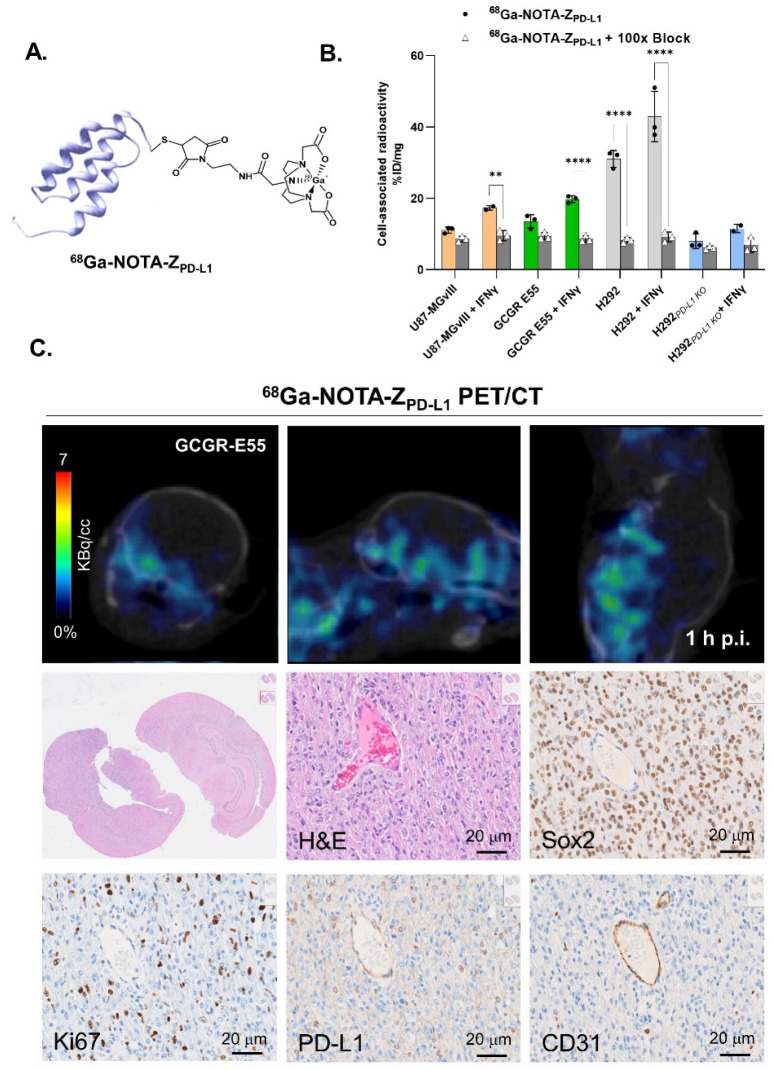
(**A**) Schematic illustrating the gallium-68-radiolabelled affibody molecule structure. (**B**) In vitro binding specificity of ^68^Ga-NOTA-Z_PD-L1_ (5 nM), with and without IFN γ stimulation as well as blocking (n = 3). **, *p* = 0.0064, ****, *p* ≤ 0.0001. (**C**) PET/CT and T_2_w MRI scans of intracranial injections of ^68^Ga-NOTA-Z_PD-L1_ 1 h postinjection in GCGR-E55 orthotopic tumours. The mice received 0.73–1.55 MBq of radioconjugate (n = 6). IHC staining of H&E, Sox2, Ki67, PD-L1 and CD31.

The ability of ^18^F-AlF-NOTA-Z_PD-L1_ and ^68^Ga-NOTA-Z_PD-L1_ to visualise PD-L1+ cells in the brain was evaluated using intracranial U87-MGvIII and GCGR-E55 tumour models, respectively (Figure 4A and Figure 5C). PET/CT images acquired 1 h post-radioconjugate injection displayed focal and superior accumulation of ^18^F-AlF-NOTA-Z_PD-L1_ in U87-MGvIII tumours that were earlier defined with T2-weighted MRI (Figure 4A). The histopathological examination of axial brain sections performed at the end of this study verified the presence of well-defined tumour masses (Figure 4B—H&E). Moreover, the analysis of PET data showed only negligible uptake of the radioconjugate in the healthy brain parenchyma, resulting in high tumour-to-background contrast images. The ROI-derived radioactivity concentration within the tumours was in the range of 1.6 ± 0.7–2.7 ± 0.7%ID/g (Figure 4C).

Conversely, ^68^Ga-NOTA-Z_PD-L1_ PET imaging of GCGR-E55 showed a widespread brain uptake pattern reflecting the dispersed nature of these particular GBM tumours. (Figure 5C, top row). The images were corroborated with subsequent H&E staining of the brain tissue showing diffuse and infiltrating growth of GCGR-E55 cells involving contiguous lobes of the brain that resemble gliomatosis cerebri (Figure 5C). In addition, there was a high abundance of cells across the entire brain displaying an increased expression of glioma stem cell marker SOX2 (Figure 5C, middle row). The proliferation marker Ki67 showed distinct and clear nuclear staining of proliferating U87-MGvIII and GCGR-E55 tumour cells Figure 4B and Figure 5C). IHC results with anti-CD31 antibody confirmed the presence of tumour-associated mouse blood vessels and, qualitatively, showed fewer CD31-positive structures in GCGR-E55 tumours than U87-MGvIII. Furthermore, both tumour types displayed high PD-L1 expression levels in the cancer cells. 

### 3.5. Distribution Pattern of PD-L1 in Human Glioma Samples

**Figure 6:**
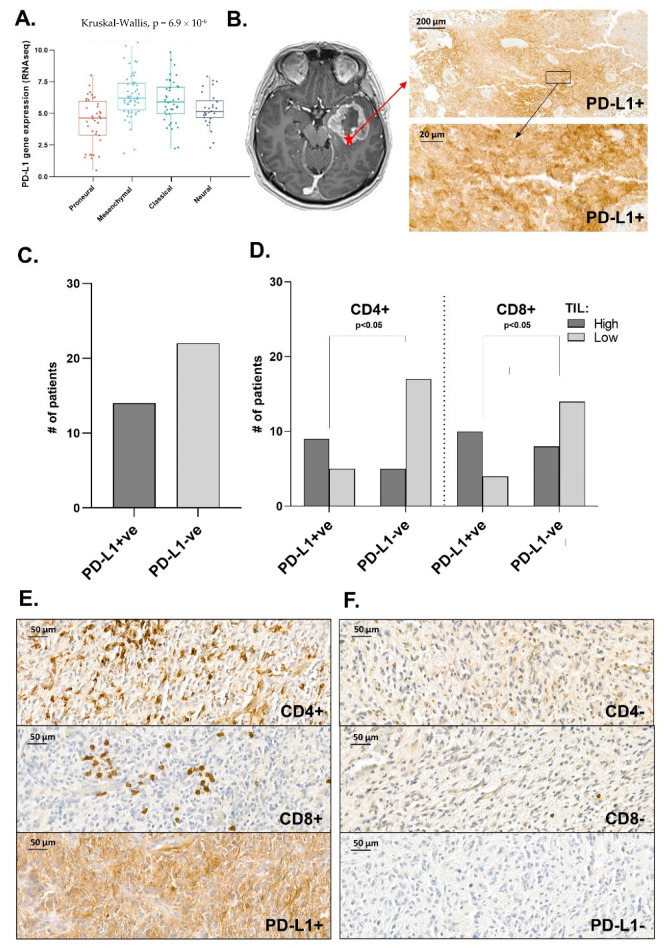
(**A**) Analysis of TCGA showing differential PD-L1 mRNA expression between GBM subtypes. (**B**) Gadolinium-enhanced T1-weighted axial image of GBM. The heterogeneous irregular peripheral enhancement is associated with the left temporal lobe mass with a central non-enhancing area, consistent with necrosis (left). IHC anty-PD-L1 staining showing membrane and cytoplasmic PD-L1 expression (right). (**C**–**E**) PD-L1, CD4 and CD8 evaluation in intraoperatively resected tumour samples (n = 36). (**F**) Representative IHC staining patterns of CD4+/CD4−, CD8+/CD8− and PD-L1+/ PD-L1− cells (original magnification ×40, scale bar 50 µm).

We analysed sequencing (RNA-Seq) data sets of GBM patients from The Cancer Genome Atlas (TCGA; n = 161 GBM samples). A Kruskal–Wallis test showed a significant (*p* < 0.05) change in the expression levels of PD-L1 in different subtypes of GBM, with the mesenchymal showing an elevated expression level of PD-L1 compared to other groups (Figure 6A). In our own data set (n = 36, newly diagnosed GBM), PD-L1 was differentially expressed. The majority of specimens showed both membrane and cytoplasmic staining (Figure 6B). PD-L1-positive membrane staining (IHC score ≥1 in at least 5% of the cells) was observed in 39% of individuals (Figure 6C). In the remaining 61% of tumour specimens, we observed only faint cytoplasmic PD-L1 expression (17%) or no expression at all (44%) (Figure 6C). Moreover, PD-L1-positive specimens showed significantly greater CD4+ and CD8+ cell infiltration (64% and 56%, respectively; *p* < 0.05) (Figure 6D). The analysed images showed the perivascular and intratumoural distribution of CD8+ and CD4+ cells (Figure 6E,F). 

## 4. Discussion

Tumoural and microenvironmental heterogeneity, both between patients and within individual tumours, is one of the leading causes of GBM therapy failure [24]. However, with the latest success of immunotherapy in other cancers, a number of clinical studies investigating the combination of PD-1/PD-L1 inhibitors with standard-of-care therapies for GBM patients have been actively pursued. 

Unfortunately, the lack of durable responses combined with the high costs of these trials have highlighted a need for the development of biomarkers that could improve the accuracy of these studies by ensuring the right patients are enrolled [25]. Interestingly, several studies have clearly demonstrated that screening specific GBM subgroups may lead to improved response rates [26]. Of note, among the three identified molecular subtypes of GBM—classical, proneural and mesenchymal—the last subtype occurs in about 30–49% of cases and manifests the worst survival rate [27,28,29]. Furthermore, GBMs originally presenting as the classical or proneural subtype may transform, due to a phenotypic shift, into the mesenchymal subtype in a manner analogous to epithelial–mesenchymal transition [30]. Most importantly, tumours with mesenchymal features, as recently reported, have elevated levels of PD-L1 and, therefore, may be more amenable to PD-1/PD-L1 checkpoint blockade [9]. 

Studying the data set from TCGA, we found differential PD-L1 expression between GBM molecular subtypes, with the highest mRNA expression level of PD-L1 in the tumours belonging to the mesenchymal subtype. Moreover, analysis of our own patient samples revealed PD-L1-positive membrane staining in 39% of GBM tumours. Interestingly, these specimens were also associated with increased CD8+ and CD4+ T cell infiltration. Of note, it has been previously demonstrated that tumour-infiltrating cytotoxic T lymphocytes can secrete IFNγ and enhance PD-1 expression, leading to the upregulation of PD-L1 in tumour cells [31,32]. However, the opposite results showing the negative correlation between the PD-L1 expression and CD8+ TIL infiltration were also reported [33,34]. Therefore, a better understanding of the GBM immune environment may be a decisive step towards the selection of patients that could most benefit from the delivery of drugs targeting the PD-1/PD-L1 axis. 

In the clinic, PD-L1 expression is routinely measured with IHC. There are four PD-L1 antibodies (i.e., 22C3, 28-8, SP263, SP142) registered with the FDA for this purpose, on two different IHC platforms (Dako and Ventana), each with their own scoring systems. Data from clinical trials have shown that PD-L1 expression, measured with tumour proportion score (TPS) or combined positivity score (CPS), is a relevant biomarker for the selection of patients and response to ICPIs for some cancers. However, the predictive effect of PD-L1 expression on the efficacy of ICIs in GBM remains debatable. For example, the Checkmate 143 clinical trial indicated that the expression of PD-L1 on tumour cells does not correlate with the efficacy of immunotherapy, which might be due to the difficulties with PD-L1 detection methods and tumour heterogeneity. Currently, it is well recognised that immune checkpoint targets are highly dynamic, and a better understanding of the spatiotemporal dynamics, which is unachievable with a single-lesion biopsy, may be critical for the development of effective therapeutic regimens. This underscores the potential of using immuno-PET imaging to noninvasively assess the distribution of PD-L1 spatially and temporally.

Against this background and to overcome the above-mentioned shortcomings, we have investigated the ability of targeted-PET imaging to measure PD-L1 expression quantitively in preclinical GBM models, providing more evidence for this approach to be translated into clinical trials for patient stratification. It has been previously reported that PD-L1-positive subcutaneous LOX melanoma tumours are strongly PET-avid for both ^18^F-AlF-NOTA-Z_PD-L1_ and ^68^Ga-NOTA-Z_PD-L1_ affibody-based radioconjugates, whereas PD-L1-negative SUDHL6 lymphoma tumours show minimal uptake [12,35]. 

Along the same lines, we successfully radiolabelled Z_PD-L1_ with fluorine-18 and gallium-68, producing high-purity products. The ability to radiolabel the same conjugate with both radioisotopes overcomes issues with regards to radionuclide availability (e.g., access to Ga-68 generator or an on-site cyclotron). The in vitro and in vivo studies confirmed that both radioconjugates recognise PD-L1-positive GBM cells with high affinity and specificity, independently from the isotope. The measured value of K_d_ was comparable to the affinity of previously reported radiolabelled affibody molecules targeting PD-L1, EGFR and HER2 [18,19,35]. In contrast to the already published preclinical studies, which have focused on PD-L1 imaging of subcutaneous tumours, we aimed to investigate whether Z_PD-L1_-based radioconjugates have the capacity to visualise PD-L1-positive cells in brain tumours. A growing body of evidence shows that tumours are known to compromise the integrity of the BBB, resulting in structural changes including neuronal death, astrocyte endfeet displacement, and heterogeneous pericyte and astrocyte subpopulations, all of which can lead to the nonuniform permeability and active efflux of molecules [36]. Until now, only a few radiotracers have been employed for the imaging of brain tumours in humans. Niemijer et al. have recently shown accumulation of anti-PD-1 mAb (^89^Zr-DFO-Nivolumab) in the brain metastases in two patients, but with lower SUV values compared to extracerebral lesions, due to low CNS penetration of the radioconjugate [37]. Additionally, ^89^Zr-CD8-specific, one-armed mAb and ^89^Zr-pemrolizumab have been successfully applied to image CD8 and PD-1 in melanoma brain metastasis [38,39]. For this purpose, the favourable energy profile and short half-life of F-18 and Ga-68, combined with the high-target specificity and favourable kinetics of affibody molecules, may allow for sequential imaging and increase the chance of capturing spatiotemporal changes in PD-L1 expression, particularly shortly after treatment initiation.

Our first in vivo data revealed high and specific tumour uptake of ^18^F-AlF-NOTA-Z_PD-L1_ in mice bearing subcutaneous PD-L1-positive tumours (U87-MGvIII) and very low nonspecific uptake in PD-L1-negative tumours (H292_PD-L1KO_). These findings were further confirmed with IHC of tumour sections performed postimaging. The kidney retention of the radioactivity observed in the PET images and biodistribution data was due to renal clearance of the affibody and tubular reuptake of the protein, as well as cleavage of the AlF complex from the affibody ligand [12]. Apart from that, the radioconjugate cleared quickly from the muscles and blood, resulting in high tumour-to-background ratios. There were very low signals in PD-L1-expressing normal organs, such as the spleen and lymph nodes, due to the lack of human-PD-L1-specific Z_PD-L1_ cross-reactivity with murine PD-L1. Therefore, radioactivity accumulation in these tissues could not be directly analysed and, in clinical practice, will require further reassessment. Most importantly, when evaluating ^18^F-AlF-NOTA-Z_PD-L1_ and ^68^Ga-NOTA-Z_PD-L1_ in the brain setting, we found that both radioconjugates accumulate rapidly in GBM brain tumours. High tumour-to-brain parenchyma contrast images were acquired as early as 1 h postadministration. 

It should be emphasised that whole-body ^18^F-AlF-NOTA-Z_PD-L1_ and ^68^Ga-NOTA-Z_PD-L1_ PET will not distinguish between the different types of cells expressing PD-L1, including GBM cells and immune cells (e.g., T cells, macrophages, dendritic cells and neutrophils). However, both radioconjugates will provide real-time quantitative information about intratumoural, as well as systemic, PD-L1 expression levels at baseline and over time in response to ICPIs. 

## 5. Conclusions

Both ^18^F-AlF-NOTA-Z_PD-L1_ and ^68^Ga-NOTA-Z_PD-L1_ showed selective accumulation in PD-L1-positive tumours and detected GBM brain tumours with high contrast. Therefore, anti-PD-L1 affibody-based immuno-PET holds great potential for noninvasive stratification of GBM patients and optimisation of PD-1/PD-L1 checkpoint blockade therapy, offering a viable remedy to the drawbacks of conventional techniques such as biopsy and IHC staining.

## Data Availability

Apart from the data presented in this paper, there were no other data generated.

## Supplementary Materials

The following supporting information can be downloaded at: https://www.mdpi.com/article/10.3390/cancers15123131/s1, Figure S1: Survival curve of intracranially implanted E55 tumours. Figure S2: Representative chromatogram of NOTA-Z_PD-L1_. Figure S3: Representative chromatograms of the radiolabelling reaction of ^18^F-AlF-NOTA-Z_PD-L1._ Figure S4: Representative chromatograms of the radiolabelling reaction of ^68^Ga-NOTA-Z_PD-L1_. Figure S5. Representative whole-body PET/CT images of mouse bearing U87-MGvIII tumour, acquired 1 h after ^18^F-AlF-NOTA-Z_PD-L1_ administration. References [22,40,41] are cited in Supplemental Data.
