# Supplementary material for: Immuno-PET Imaging of Tumour PD-L1 Expression in Glioblastoma
Source: Cancers (Basel). 2023 Jun 9;15(12):3131. doi: 10.3390/cancers15123131 (PMC10295898; doi:10.3390/cancers15123131)
Supplement: Supplementary file 1 [file cancers-15-03131-s001.zip › cancers-2408012-supplementary.pdf]

Immuno-PET Imaging of Tumour PD-L1 Expression in Glioblastoma

Supplemental Data

Authors: Gitanjali Sharma[1], Marta C. Braga[1], Chiara Da Pieve[1], Wojciech Szopa[2], Tatjana Starzetz[3,4], Karl Heinz Plate[3,4], Wojciech Kaspera[2], Gabriela Kramer-Marek[1*]

[1] *Institute of Cancer Research, Department of Radiotherapy and Imaging, London, United Kingdom*

[2] *Medical University of Silesia, Department of Neurosurgery, Katowice, Poland*

[3] *Edinger Institute, Institute of Neurology, Frankfurt, Germany*

[4] *German Consortium for Translational Cancer Research, DKTK, Heidelberg, Germany*

Contents:

Materials and Methods

Figure S1. Survival curve of intracranially implanted E55 tumours.

Figure S2. Representative chromatogram of NOTA-Z_{PD-L1}.

Figure S3. Representative chromatograms of the radiolabelling reaction of ^{18}F-AlF-NOTA-Z_{PD-L1}.

Figure S4. Representative chromatograms of the radiolabelling reaction of ^{68}Ga-NOTA-Z_{PD-L1}.

Figure S5. Representative whole-body PET/ CT images of mouse bearing U87-MGvIII tumour, acquired 1 h after ^{18}F-AlF-NOTA-Z_{PD-L1} administration.

References

MATERIALS AND METHODS

General

All the reagents and solvents were purchased from commercial sources and used without further purification unless otherwise stated. NOTA-Z_{PD-L1}, prepared by attaching NOTA-maleimide to the terminal cysteine on the affibody molecule against human PD-L1 Z_{PD-L1}, was kindly provided by GE Healthcare Limited (Buckinghamshire, UK). *High-performance liquid chromatography* (HPLC) grade acetonitrile and trifluoroacetic acid (TFA), dimethyl sulfoxide (DMSO) and ethanol (EtOH) were purchased from Thermo Fisher Scientific (Loughborough, UK). Phosphate buffered saline (PBS) was purchased from Gibco (Thermo Fisher Scientific, Paisley, UK). Mouse serum and Iso-Disc PVDF syringe filters (13 mm, 0.2 μm) were purchased from Sigma-Aldrich (Gillingham, UK). Aluminium chloride hexahydrate ($AlCl_3{\cdot}6H_2O$, 99.9995%) was purchased from Alfa Aesar (Heysham, UK). Sodium acetate (AnalR Normapur) was purchased from VWR International (Lutterworth, UK). Low protein-binding microcentrifuge tubes (1.5 mL) were purchased from Eppendorf (Stevenage, UK). Incubation of the reaction mixtures was performed using a Grant Bio thermo-shaker (Camlab, Stevenage, UK). Protein concentration was determined by measuring the UV absorbance at 280 nm on a NanoDrop 2000 spectrophotometer (Thermo Fisher Scientific, Loughborough, UK) using 15340 $M^{-1}cm^{-1}$ as the molar extinction coefficient. Oasis HLB solid phase extraction (SPE) cartridges (1 mL, 30 mg sorbent) were purchased from Waters (Elstree, UK) and chromabond strong cation-exchange SPE cartridges (1 mL, 100 mg sorbent, Macherey-Nagel) were supplied by Greyhound Chromatography (Wallasey, UK). [^{18}F]Fluorine, produced on a GE PETrace cyclotron by 16 MeV irradiation of an enriched [^{18}O]H_2O target, was supplied by Alliance Medical Radiopharmacy Ltd (Warwick, UK) and used without further purification. ^{68}Gallium in 0.1 M hydrochloric acid from a Galli Ad ^{68}Ge/^{68}Ga generator (IRE ELiTE Radiopharma, Fleurus, Belgium) was supplied by the Royal Marsden Hospital radiopharmacy (Sutton, UK). Reverse phase high-performance liquid chromatography (RP-HPLC) was carried out on an Agilent Infinity 1260 quaternary pump system equipped with a 1260 Diode array (Agilent Technologies, Didcot, UK). Elution profiles were analysed using Laura software v.4.2.6.79 (Lablogic, Sheffield, UK). Affibody conjugates and radioconjugates were analysed on a Zorbax-300SB C18 column, 4.6×250 mm, 5 μm (Agilent Technologies, Didcot, UK) using the following gradient: 0-12 min. 23%-35% B, 12.1-15 min. 35%-50% B with 0.1% TFA in water as eluent A and 0.1% TFA in acetonitrile as eluent B at a flow rate of 1 mL/min.

Preparation of ^{18}F-AlF-NOTA-Z_{PD-L1}

To a 1.5 mL low protein microcentrifuge tube, NOTA-Z_{PD-L1} in 0.5 M sodium acetate pH 4 (12 nmol), 2 mM $AlCl_3$ (10 nmol) in 0.5 M sodium acetate pH 4, and aqueous non-purified ^{18}F-fluoride (150-180 MBq) were added followed by an equal volume of ethanol/DMSO mixture (1:1 v/v). The solution was incubated at 95 °C for 15 min. After cooling to ambient temperature, the solution was purified by RP-HPLC. The fraction containing the product was collected, diluted with 0.1% aqueous TFA (3 mL) and loaded onto the hydrophilic-**l**ipophilic **b**alance cartridge (Oasis HLB-SPE). The cartridge was washed with 0.1% aqueous TFA (5 mL) followed by water (3 mL). The product was eluted with 50% ethanol/water (v/v, approximately 130 μL). The protein concentration was measured by UV at the wavelength of 280 nm. Preparation time = ca. 50 min.

Preparation of ^{68}Ga-NOTA-Z_{PD-L1}

The eluate from a ^{68}Ge/^{68}Ga generator in 0.1 M HCl (1.1 mL) was loaded onto a chromabond-SPE cartridge preconditioned with 0.1 M HCl. [$^{68}GaCl_4$] was then eluted from the cartridge using a 5 M NaCl/5.5 M HCl solution (300 μL) and the pH of the eluate was adjusted to 4 using 0.2 M sodium acetate buffer (pH 6, 420 μL). An aliquot of the buffered ^{68}Ga solution (ca. 220 MBq) was added to NOTA-Z_{PD-L1} (8 nmol) in a 1.5 mL low-protein-binding microcentrifuge tube and the mixture was incubated at 40

°C for 15 min. A solution of 0.1 M EDTA was added to the reaction mixture which was then loaded onto an Oasis HLB-SPE cartridge. The cartridge was washed with 0.1% aqueous TFA (3 mL) followed by water (3 mL). ^{68}Ga-NOTA-Z_{PD-L1} was eluted from the HLB cartridge with an ethanol-water solution (1:1 v/v, approximately 150 μL). The protein concentration was determined by measuring the UV absorbance at 280 nm. Preparation time = ca. 40 min.

Cell lines and tumours

Human non-small cell lung cancer cell lines (H292 and $H292_{PD-L1KO}$) were provided by the Medical Oncology Laboratory, University Medical Center, Groningen (UMCG) and cultured in RPMI (Gibco, Thermo Fisher Scientific, Paisley, UK) supplemented with 10% foetal bovine serum (FBS) (Gibco). Human glioblastoma (U87-MGvIII) cell line was provided by Dr Frank Furnari (Ludwig Cancer Research, San Diego, USA) and cultured in DMEM medium (Gibco) supplemented with 10% FBS and 400 μg/mL of selective antibiotic (Geneticin™, G418 Sulfate, Thermo Fisher Scientific, Loughborough, UK). The glioma stem cell line (GCGR-E55) was provided by Prof. Steven Pollard (University of Edinburgh, UK). Cells were cultured in DMEM/Nutrient Mixture F-12/Ham medium supplemented with glucose, MEM NEAA, BSA, 2-mercaptoethanol, N2 and B27 (all from Thermo Fisher Scientific, Loughborough, UK), mouse EGF, human FGF (both from PeproTech, Cranbury, US) and laminin (Cultrex® 3D Culture Matrix Laminin I, Trevigen, Gaithersburg, US). All cells were kept in a humidified chamber at 37° C supplied with 5% CO_2 and used until passage ten. Polymerase chain reaction tests were used to confirm negative mycoplasma results (Surrey Diagnostics, Cranleigh, UK). Also, the genetic origin of the cell lines was tested and authenticated by short tandem repeat (STR) DNA profiling analysis (Eurofins Medigenomix, Germany). TCGA RNA Seq data were obtained from the TCGA Data Portal (http://cancergenome.nih.gov). Tissue specimens from the patients with newly diagnosed GBM (n = 36) were collected for IHC in accordance with the protocol approved by the Institutional Bioethical Committee of Medical University of Silesia (Katowice, Poland). Consent was given by all the patients. The fresh tissues, surgically removed from patients were fixed in formalin and histologically processed for paraffin-embedded blocks (FFPE).

Flow analysis of PD-L1 expression

To assess the PD-L1 expression in H292 (PD-L1ve++), U87-MGvIII, GCGR-E55 (PD-L1ve+) and $H292_{PD-L1KO}$ (PD-L1ve-) cell lines, the cells (2.5×10^5 cells/sample) were washed once with cold PBS and incubated for 40 mins at 4° C with either a specific anti-CD274 PD-L1 anti-human antibody (MIH1, PE Cyanine-7, 25-5983-42, Invitrogen, Waltham, USA**)** or PBS (for the unstained controls). Subsequently, the cells were washed twice with cold PBS and then resuspended in PBS (0.5 mL) for data acquisition. Flow cytometry was performed using a BD LSRII flow cytometer (BD, Biosciences, Swindon, UK) and during each run a total of 10,000 events per sample were recorded. All viable, single cells were gated, plotted and analysed using FlowJo v10 (BD, Biosciences). The difference of mean fluorescence intensity (MFI) between stained and unstained samples was used to quantify the PD-L1 expression in cells.

^{18}F-AlF-NOTA-Z_{PD-L1} and ^{68}Ga-NOTA-Z_{PD-L1} *in vitro* studies

Specificity of binding: For the study using ^{18}F-AlF-NOTA-Z_{PD-L1}, H292, U87-MGvIII, GCGR-E55 and $H292_{PD-L1KO}$ cells were seeded on 24-well plates (Thermo Fisher Scientific) (3×10^5 cells/well) 48 h prior to the experiment. For the study using ^{68}Ga-NOTA-Z_{PD-L1}, H292 and $H292_{PD-L1KO}$ cells (3×10^5 cells/well), U87-MGvIII (4×10^5 cells/well) and GCGR-E55 (6×10^5 cells/well) were seeded on 24-well plates 48 h prior to the experiment. One group of cells was stimulated with human interferon gamma (IFNγ,20 ng/mL, PHC4031, Gibco, Thermo Fisher Scientific) overnight whilst the rest (control cells) were incubated with their respective media only. All cells were then washed twice with PBS and incubated with a 5 nM solution of either ^{18}F-AlF-NOTA-Z_{PD-L1} (20-30 kBq/well) or ^{68}Ga-NOTA-Z_{PD-L1} (15 kBq/well). For blocking experiments, cells were pre-incubated with 100-fold molar excess of non-labelled affibody conjugate (NOTA-Z_{PD-L1}) for 10 min before adding the radioconjugate. After 1 h, the supernatant was removed, cells were rinsed twice with cold PBS and trypsinised. Cell-associated and decay-corrected radioactivity was determined by γ-counter (Wizard^2 2480, PerkinElmer, Beaconsfield, UK). The data

were expressed as a percentage of the incubated dose (%ID) per mg of the protein lysate. The protein concentration of the lysates was determined by the BCA assay (Pierce, Waltham, USA).

Saturation radioligand binding assay: To determine the dissociation constant (K_d) of ^{18}F-AlF-NOTA-$Z_{PD\text{-}L1}$, H292 cells were seeded on 24-well plates (3×10^5 cells/well) 24 h prior to the study and grown to a monolayer confluency. On the following day, cells were incubated with increasing concentrations of ^{18}F-AlF-NOTA-$Z_{PD\text{-}L1}$ (0.01 nM - 15 nM, 0.45-50 kBq/well) in non-supplemented RPMI media for 1 h at 4°C. Non-specific binding was determined through co-incubation of 100-fold molar excess of non-radiolabelled affibody conjugate. The cells were then rinsed three times with cold PBS, trypsinised and collected into vials for radioactivity measurement by γ-counter. The K_d was estimated by plotting the amount of bound (nM) vs free radioconjugate ligand (nM). The specific binding was calculated by subtracting the non-specific binding data points from the total binding and fitted to a one-site receptor-binding model using GraphPad Prism 9 (San Diego, USA).

Mouse Models

All experiments were performed in compliance with the licence issued under the UK Animals (Scientific Procedures) Act 1986, the UK National Cancer Research Institute Guidelines for Animal Welfare in Cancer Research (*21*) and the ARRIVE guidelines for reporting animal research. All experiments were conducted under the Project Licence PPL PCC916B22, approved by the UK Home Office and by the local ethical review committee. Female nude mice (crl:NU(NCR)-Fox1n1nu, 6-7 weeks old) and female NSG (NOD.Cg-Prkdc^{scid} Il2rgtm1Wjl/SzJ) were obtained from Charles River Laboratories (Harlow, UK). Animals were housed in individually ventilated cages (Allentown Nexgen, New Jersey, US) with an area of 500 cm^2 and a maximum of 5 animals per cage, temperature of 21±1°C, humidity of 55±10% and 12:12 h of dark:light cycles, and given food (Labdiet, St. Louis, US) and water *ad libitum.* Environmental enrichment included bedding substrate of Corn Cob 6/8, Aspen chew stick 5×1×1 cm (Datesand, Stockport, UK) and one Bed r'nest paper wool nesting material (Datesand, Stockport, UK).

For subcutaneous models, animals (n = 4 per group) were injected over the right shoulder with U87-MGvIII cells (1.5×10^5 in 70 μL PBS with 30% Matrigel™ (BD Bioscience) or H292$_{PD\text{-}L1\ KO}$ cells (8×10^6 in 100μl PBS with 50% Matrigel™). For orthotopic implantation, animals (n = 6-8 per group) were anaesthetised with isoflurane (3-4% induction, 1-2% maintenance v/v in O_2 at 1 L/min) and stereotactically injected with U87-MGvIII cells (1.5×10^5 in 3 μl PBS) or with GCGR-E55 cells (2 ×10^5 in 2 μL PBS) cells. The injection site was located at 2 mm lateral to the sagittal suture, 1 mm anterior to the coronal suture, and 3.2 mm deep from the surface of the skull. Orthotopic tumour growth was monitored by 1 T M3™ MRI system (Aspect Imaging, Israel). Mice bearing U87-MGvIII tumours were scanned once the tumours reached 2-3 mm in the larger diameter (9-14 days post-implantation). Mice bearing GCGR-E55 tumours were imaged 18-20 weeks post-implantation based on their survival curve (Supplementary Fig. S1). Humane endpoints were specified for any neurological signs of disease or weight loss exceeding 15% of baseline bodyweight. Subcutaneous tumour growth was monitored through calliper measurements twice per week and the volume was estimated through the ellipsoid formula $V = height\ (h)\ \times width\ (w) \times length\ (l) \times {}^{\pi}/_{6}$. Animals were imaged once tumours reached approximately 70 mm^3 (1 week after injection of H292$_{PD\text{-}L1KO}$ cells and 2 weeks after injection of U87-MGvIII cells). Humane endpoints were specified for tumours exceeding a mean diameter of 12 mm.

^{18}F-AlF-NOTA-$Z_{PD\text{-}L1}$ and ^{68}Ga-NOTA-$Z_{PD\text{-}L1}$ *in vivo* imaging and *ex vivo* studies

For PET studies, animals were anaesthetised with isoflurane (3-4% induction, 1-2% maintenance v/v in O_2 at 1 L/min) and injected with ^{18}F-AlF-NOTA-$Z_{PD\text{-}L1}$ (0.5 μg, 0.1±0.05 MBq/mouse; or 1 μg, 0.4±0.3 MBq/mouse in 100 ◎L of 0.9% sodium chloride solution) intravenously via the tail vain. For the brain PET imaging studies, mice were either injected with ^{18}F-AlF-NOTA-$Z_{PD\text{-}L1}$ (1 μg in 100 ◎L of 0.9% sodium chloride solution, 0.2-0.76 MBq/mouse) or ^{68}Ga-NOTA-$Z_{PD\text{-}L1}$ (1 μg in 100 ◎L of 0.9% sodium chloride solution; 0.73-1.55 MBq/mouse) intravenously via the tail vein. For blocking studies, animals

were co-injected with 400 μg of the non-labelled affibody molecule. PET/CT scans were acquired 1 h post-injection using a small-animal PET scanner (Albira PET/SPECT/CT, Bruker, MA, US). Whole body static 15 min PET scans were acquired with a 358 to 664 keV energy window, followed by CT acquisition (10 min) that was used for scatter and attenuation corrections. PET images were reconstructed using a maximum-likelihood expectation-maximization (MLEN) algorithm (12 interactions) with a voxel size of 0.5×0.5×0.5 mm^3. High-resolution CT scans were performed with the X-ray tube set-up at a voltage of 45 kV, current of 400 μA, 250 projections (1 s per projection), and a voxel size of 0.5×0.5×0.5 mm^3. The CT images were reconstructed using a filtered-back-projection (FBP) algorithm. Image analysis was performed using the PMOD software package (PMOD Technologies Ltd., Zurich, Switzerland). Radioactivity uptake in the tumour was quantified by volume-of-interest (VOI) analysis and expressed as the mean (Mean) and the mean of the 50 hottest voxels ($Mean_{50}$) within the VOI. Data was expressed as percentage of the injected dose per gram of tissue (%ID/g) normalised to a calibration factor (MBq/g/counts) calculated by scanning a source (^{18}F or ^{68}Ga) of known activity and volume.

For biodistribution studies, animals were culled by cardiac puncture immediately after scanning. Blood, major organs as well as the tumours were collected, weighed and the radioactive content measured by γ-counting. The decay-corrected data were expressed as a percentage of injected dose per gram of tissue (%ID/g) (n = 4 ± SD).

***Ex vivo* immunohistochemistry**

The tumours were fixed in formalin (10%, v/v), embedded in paraffin, sectioned into 4-5 mm thick slices and mounted on glass slides. Sequential sections were stained with haematoxylin and eosin (H&E) and, for all antibodies, staining of xenografts tissues was performed on an automated Leica Bond III immunostainer (Leica, Wetzlar, Germany). The antibodies used in this study were: anti-CD31 mAb (1:20, Clone SZ31, DIA310, Dianova, Hamburg, Germany), anti-Ki67 (1:100, Clone SP6, Invitrogen, Waltham, US) and PD-L1 (Clone E1L3N, 13684, Cell Signalling Technology, London, UK). The sections were dewaxed and treated with BOND Epitope Retrieval Solution 2 (pH 9, AR9640, Leica, Wetzlar, Germany) for 10 min (CD31) or 20 min (PD-L1, Ki67) at 97 °C, followed by peroxidase blocking for 5 min at room temperature (RT). Incubation with the primary antibody was performed for 15 min at RT. For CD31 staining, sections were subsequently incubated with rabbit anti-rat antibody (1:500, 312-006-045, Dianova, Hamburg, Germany) for 20 min at RT. Slides were then incubated with goat anti-rat IgG polymer detection kit (Vector Laboratories, Burlingame, USA) for 8 min, followed by DAB (liquid DAB + substrate chromogen system, Dako, Agilent, Stockport, UK) for 10 min. Counterstaining was performed with Gill's haematoxylin (Sigma-Aldrich, Taufkirchen, Germany) for 8 min. IHC and H&E stained mouse formalin-fixed paraffin-embedded (FFPE) sections were scanned on a Vectra Polaris Automated Quantitative Pathology System (Akoya Biosciences Inc., Marlborough, US) at 0.5 μm/pixel. Whole slide image analysis was performed using Phenochart, version 1.0.12 (Akoya Biosciences Inc., Marlborough, US) and HALO image analysis software, version 2.1 (Indica Labs, Albuquerque, US).
FFPE tissue specimens from the patients were deparaffinized in xylene and hydrated by immersion in a series of graded ethanol. Antigen retrieval was performed in water bath (95 °C) by incubating the sections in a citrate-based buffer (pH 6) (Vector Laboratories) or Tris/EDTA buffer (pH 9) (Dako Agilent). Endogenous peroxidase activity was quenched by immersion in 3% hydrogen peroxide in tap water for 10 minutes at RT. Nonspecific binding of the mAbs was blocked by incubating the sections with 2.5% normal horse serum for 1 h at RT. Sections were then incubated overnight with the respective primary mAb at 4 °C (anti-PD-L1, 1:50, Clone 22C3, M3653, Dako Agilent, Carpinteria, CA, US; anti-CD4, dilution 0.083 μg/mL, clone EPR6855, ab133616, Abcam, Cambridge, UK; anti-CD8, dilution 0.175 μg/ml, Clone 144B, Abcam, Cambridge, UK). The signal was then developed with a ImmPress HRP Polymer Detection Kit (Vector Laboratories) and the sections were counterstained with Gill's Hematoxylin (Vector Laboratories). The images were acquired with an XC-30 camera coupled with BX43 optical microscope (Olympus) and digitized using an automatic Pannoramic 250 Flash III scanner with a resolution of 0.24 μm/pixel. Regions of interest (ROI) in individual scans were defined in CaseViewer version 2.3.0 (3DHistech, Budapest, Hungary) and exported as tiff files for further analysis.

PD-L1, CD4 and CD8 were evaluated using the free ImageJ software (https://imagej.nih.gov/ij). For each tumour 15 regions (squares with 200 µm sides) were selected. PD-L1 expression on tumour cells was evaluated according to the method described by Garber et al. (*33*). The membrane staining was graded semi-quantitatively as follows: 0 (no stained cells), positive when at least 5% of the cells showed ≥1+ membrane intensity. CD4 and CD8 staining was expressed as a number of positive cells per square millimetre of the ROI. If the median number of $CD4^+$ or $CD8^+$ cells were ≥6 and ≥2, respectively, the specimens were considered as highly positive (*34*).

Statistical Analysis

Statistical analysis for the *in vivo* data was performed using ordinary one-way ANOVA with Tukey's multiple comparisons tests and significance was considered for * $p<0.05$, ** $p<0.01$, *** $p<0.001$ and **** $p<0.0001$. Correlation between PET imaging and biodistribution data was performed by simple linear regression. The chi-square (χ^2) test was used to determine the statistical relationship between CD4+ and CD8+ cells distribution (high/low) and PD-L1 protein status (positive/negative). All analyses were carried out with Prism Software (Graphpad Software v9.1.1, San Diego, CA).

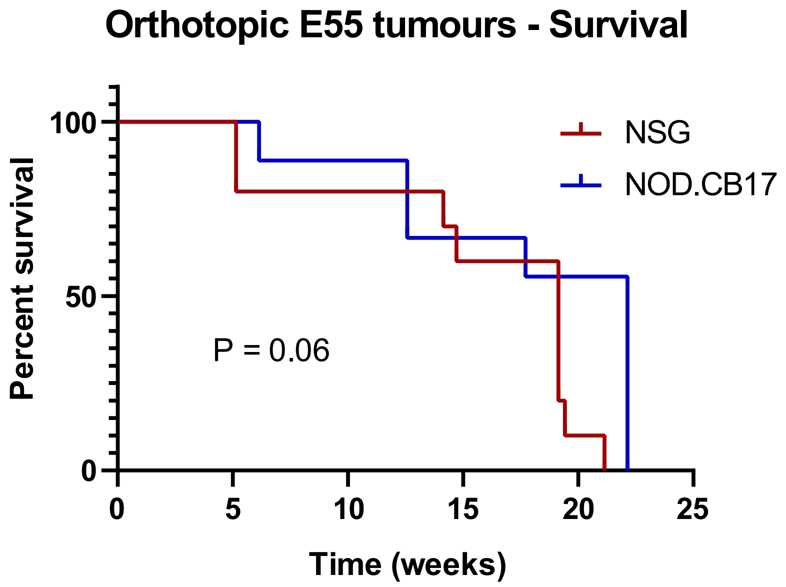

Figure S1. Survival curve of intracranially implanted E55 tumours. NSG (NOD.Cg-Prkdcscid IL2rgtm1Wjl/SzJ) (n=10) and NOD.CB17 (Prkdcscid il2rgtm1/Bc) (n=9) were injected with 250,000 and 200,00 cells, respectively, in 2 μl of PBS and at an injection rate of 1 μl/min. Injections were done at the coordinates y=+2 and x=-1 and z=-3.4 first and injection at z=-3.2 relative to the bregma. Cells were allowed to settle for 2 min before withdrawing the syringe.

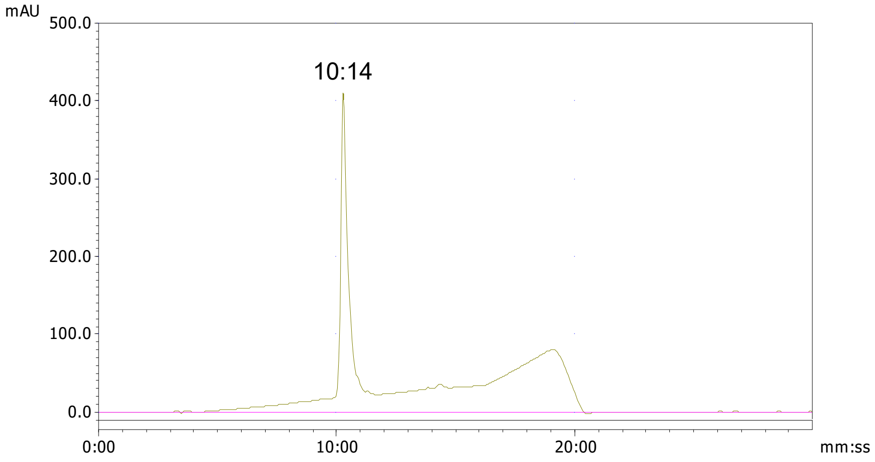

Figure S2. Representative chromatogram of NOTA-Z_{PD-L1}. The absorbance was recorded at the wavelength of 230 nm. The retention time (R_t) is indicated as minute:seconds (min:s)

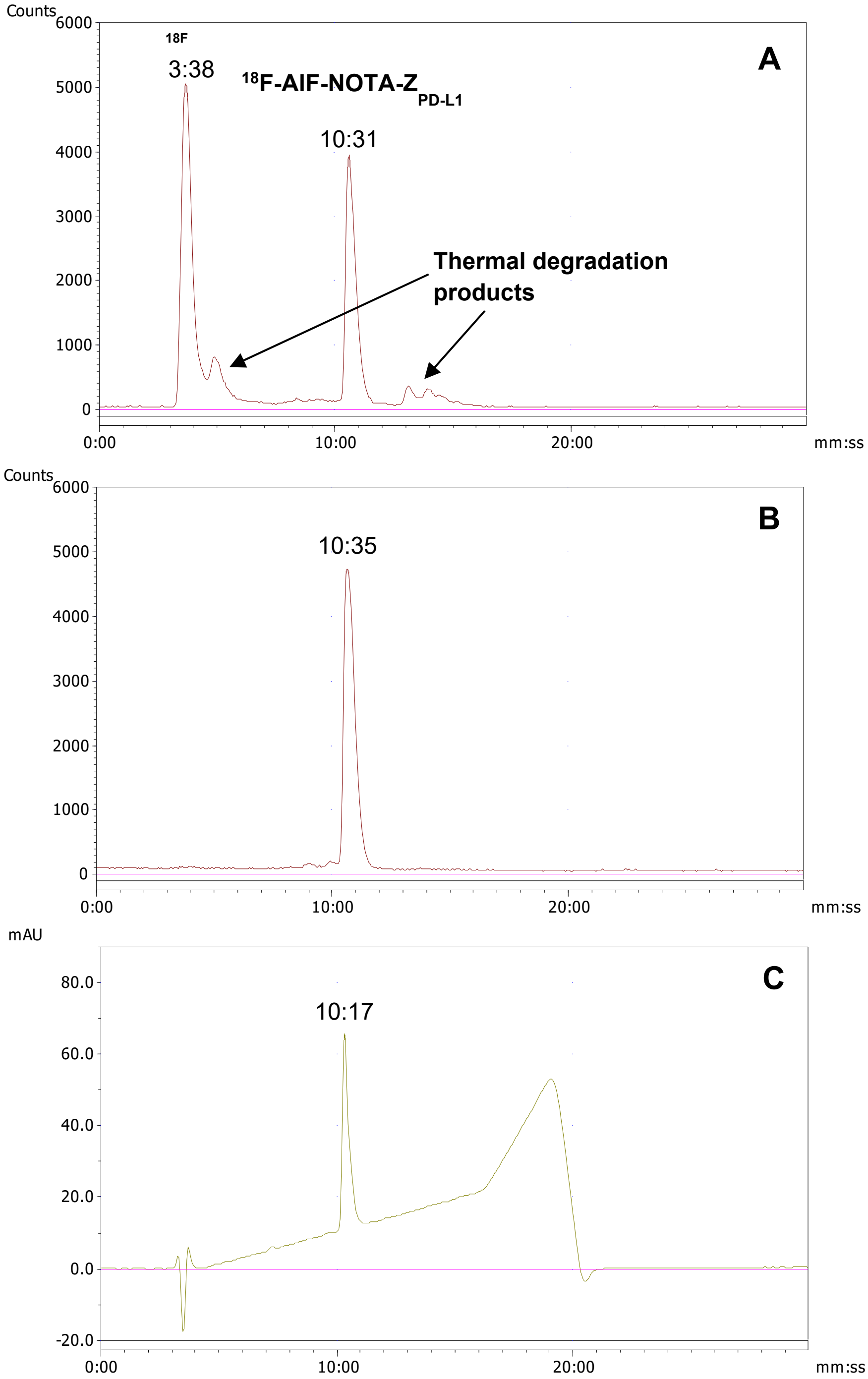

Figure S3. Representative chromatograms of the radiolabelling reaction mixture **(A)** and purified ^{18}F-AlF-NOTA-Z$_{PD-L1}$ **(B** and **C)**. The absorbance was recorded at the wavelength of 230 nm **(C)**. The retention time (R_t) is indicated as minutes:seconds (min:s)

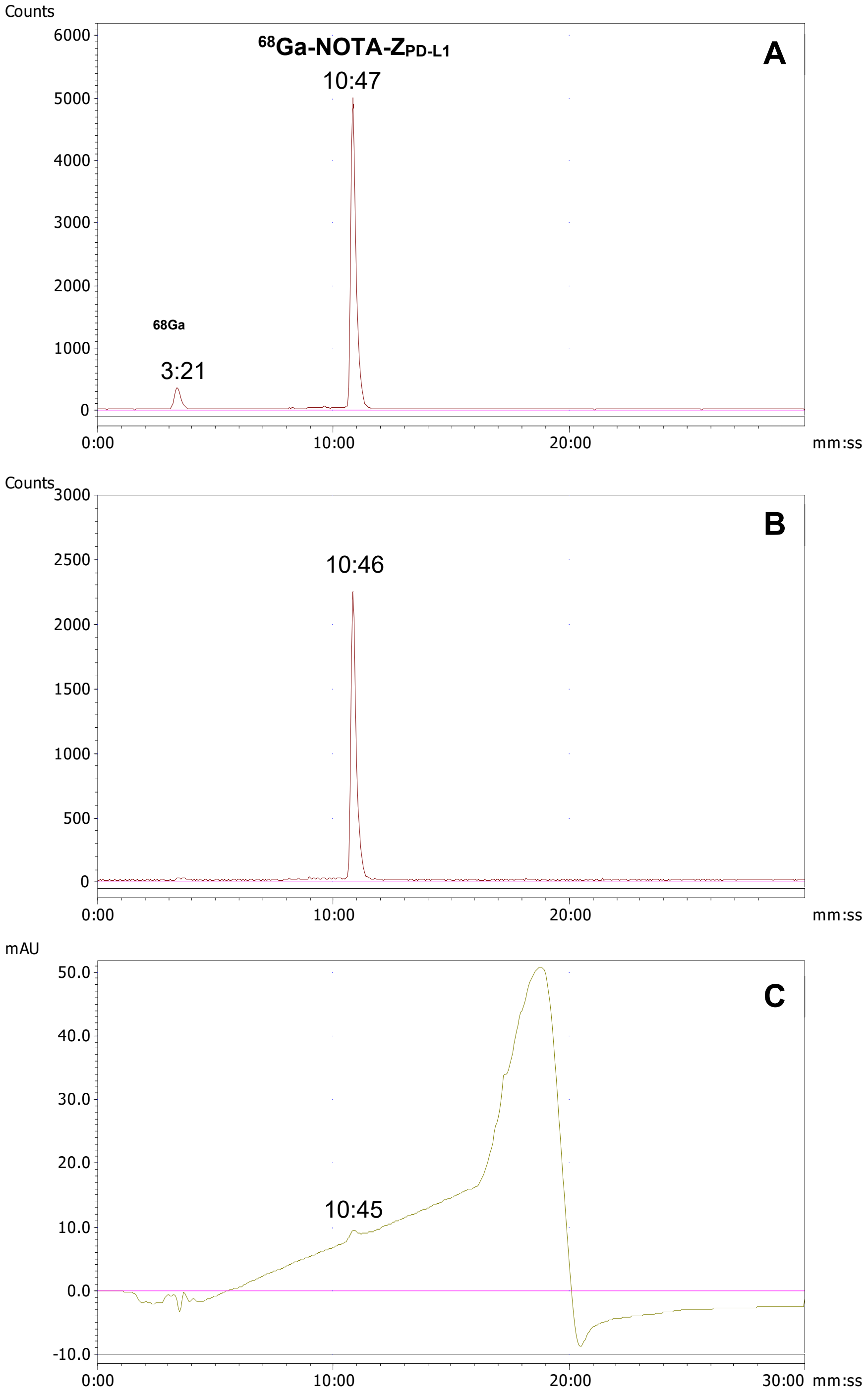

Figure S4. Representative chromatograms of the radiolabelling reaction mixture **(A)** and purified ^{68}Ga-NOTA-Z_{PD-L1} **(B and C).** The absorbance was recorded at the wavelength of 230 nm **(C)**. The retention time (R_t) is indicated as minutes:seconds (min:s)

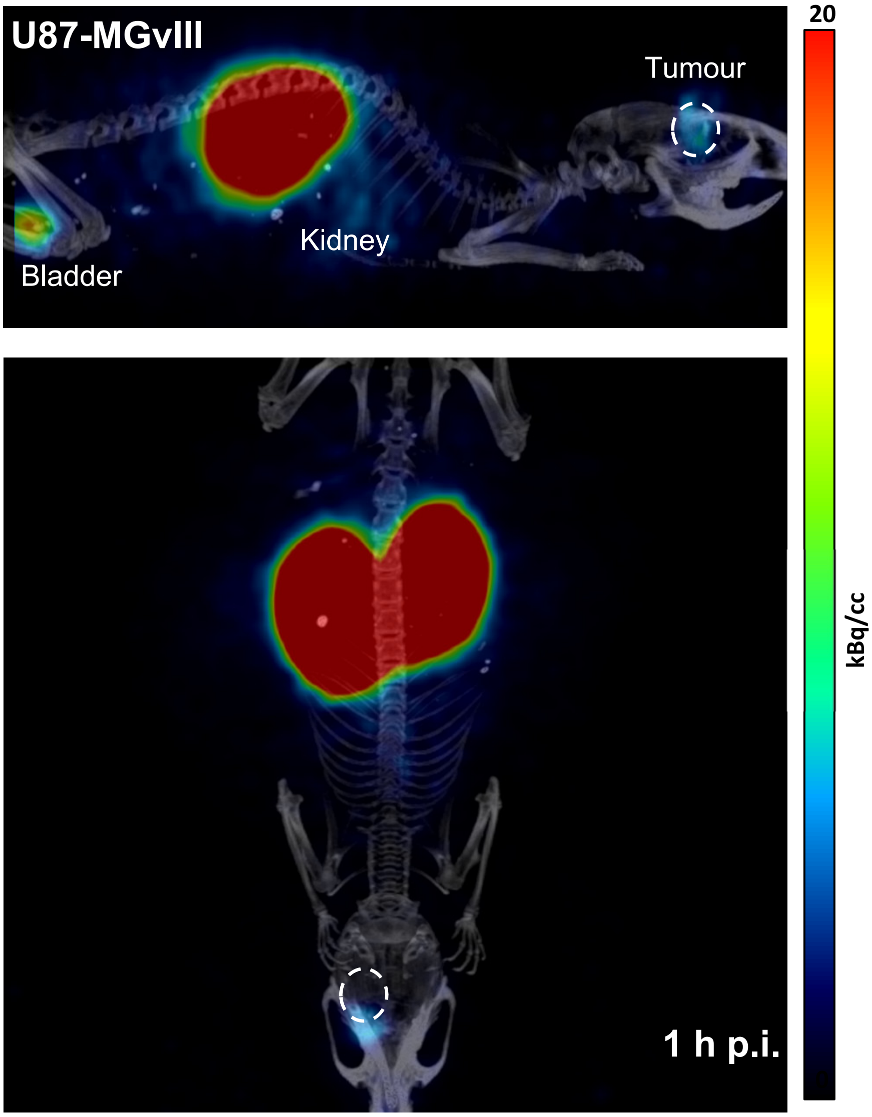

Figure S5. Representative whole-body PET/ CT images of mouse bearing U87-MGvIII tumour, acquired 1 h after ^{18}F-AlF-NOTA-Z_{PD-L1} administration.

REFERENCES

1. Workman P, Aboagye EO, Balkwill F, et al. Guidelines for the welfare and use of animals in cancer research. *British Journal of Cancer.* 2010;102:1555-1577.

2. Garber ST, Hashimoto Y, Weathers SP, et al. Immune checkpoint blockade as a potential therapeutic target: surveying CNS malignancies. *Neuro Oncol.* 2016;18:1357-1366.

3. Han S, Zhang C, Li Q, et al. Tumour-infiltrating CD4(+) and CD8(+) lymphocytes as predictors of clinical outcome in glioma. *British Journal of Cancer.* 2014;110:2560-2568.
